# An Insidious Source of Chronic Sinusitis and Recurrent Epistaxis: A Rare Case of Skull Base Glomangiopericytoma

**DOI:** 10.7759/cureus.83348

**Published:** 2025-05-02

**Authors:** Ismail Ozgenc, Alper Ozdilek, Mehmet Ozgenc, Demet Etit, Nazim Korkut

**Affiliations:** 1 School of Medicine, St George's University of London, London, GBR; 2 Department of Otolaryngology - Head and Neck Surgery, Acibadem Maslak Hospital, Istanbul, TUR; 3 Department of Otolaryngology - Head and Neck Surgery, Entodent Clinic, Nicosia, CYP; 4 Department of Pathology, Acibadem Maslak Hospital, Istanbul, TUR

**Keywords:** adult rhinology, functional endoscopic sinus surgery, sinonasal glomangiopericytoma, sinonasal tumor, sinonasal tumors invading skull base, skull base tumors, sphenoid sinusitis, tumor epistaxis

## Abstract

Glomangiopericytoma (GLC) is a rare malignant tumor of the sinonasal cavity that can lead to a variety of non-specific symptoms and consequently cause inaccurate diagnosis and delayed management. This can ultimately cause irreversible damage to the quality of life and result in a poor prognosis. We present the case of a 55-year-old male patient who suffered from the symptoms of chronic rhinosinusitis, anosmia, and multiple episodes of epistaxis with an endoscopic finding of a right-sided polyp-like mass within the sinonasal cavity. Further imaging studies revealed a mass originating from the skull base and a right-sided sphenoid sinus mucocele. Endoscopic endonasal resection and histopathological analysis confirmed a rare skull base GLC. The objective of this case report is to emphasize the diagnostic challenge of GLC due to its rare occurrence and bring awareness to its insidious and malignant nature that hides behind long-term sinonasal symptoms, mimicking chronic rhinosinusitis.

## Introduction

Sinonasal glomangiopericytoma (GLC) is a mesenchymal neoplasm with a perivascular myoid phenotype [1]. This differentiates GLC from other hemangiopericytomas as both types have a morphological characteristic, specifically, a “staghorn” vasculature [1]. Hemangiopericytoma was first described in 1942 as a soft tissue tumor with vascular branching and proliferation [2]. Later in 1998, Granter et al. introduced the term GLC to better represent its morphological features that resemble both hemangiopericytomas and glomus tumors [3]. Consequently, it was determined by the World Health Organization (WHO) in 2005 that GLC is a borderline, low malignant potential neoplasm [4]. 

Macroscopically, GLC can have a reddish or grayish-bluish, friable, and lobulated appearance [1]. The identification of nuclear β-catenin expression and CTNNB1 mutations has been pivotal in distinguishing GLC from a wide range of differential diagnoses based on its molecular profile. [1]. Definitive diagnosis is made with a histopathological assessment, and the mainstay treatment is the endonasal endoscopic excision of the mass with tumor-free margins [5,6]. In cases of incomplete resection, metastasis, or in patients unfit for surgery, radiotherapy may be considered as an alternative treatment modality [7].

GLC is a rare entity. It is responsible for less than 0.5% of all sinonasal neoplasms [8]. Only a few cases are reported in the literature, and no cases with concurrent sinus mucocele have been reported to the best of our knowledge. It can present with epistaxis and other nonspecific sinonasal symptoms such as headaches and nasal obstruction, making it a diagnostic challenge [5]. Although the most affected region is the nasal cavity, more uncommonly, GLC can originate from the sinuses and the skull base. The disease has a slight female dominance, and the peak age is shown to be 60-70 years [7].

In this report, we describe the case of a 55-year-old male patient with GLC originating from the skull base who was successfully treated with endoscopic resection.

## Case presentation

A 55-year-old male patient with a five-year history of chronic rhinosinusitis and intermittent anosmia acutely presented to the otolaryngology clinic with epistaxis. The bleeding was managed with anterior packing, and the initial investigations, including a full blood count, liver function tests, and coagulation panels, were unremarkable. The patient reported two prior episodes of epistaxis over the preceding six months. 

**Figure 1:**
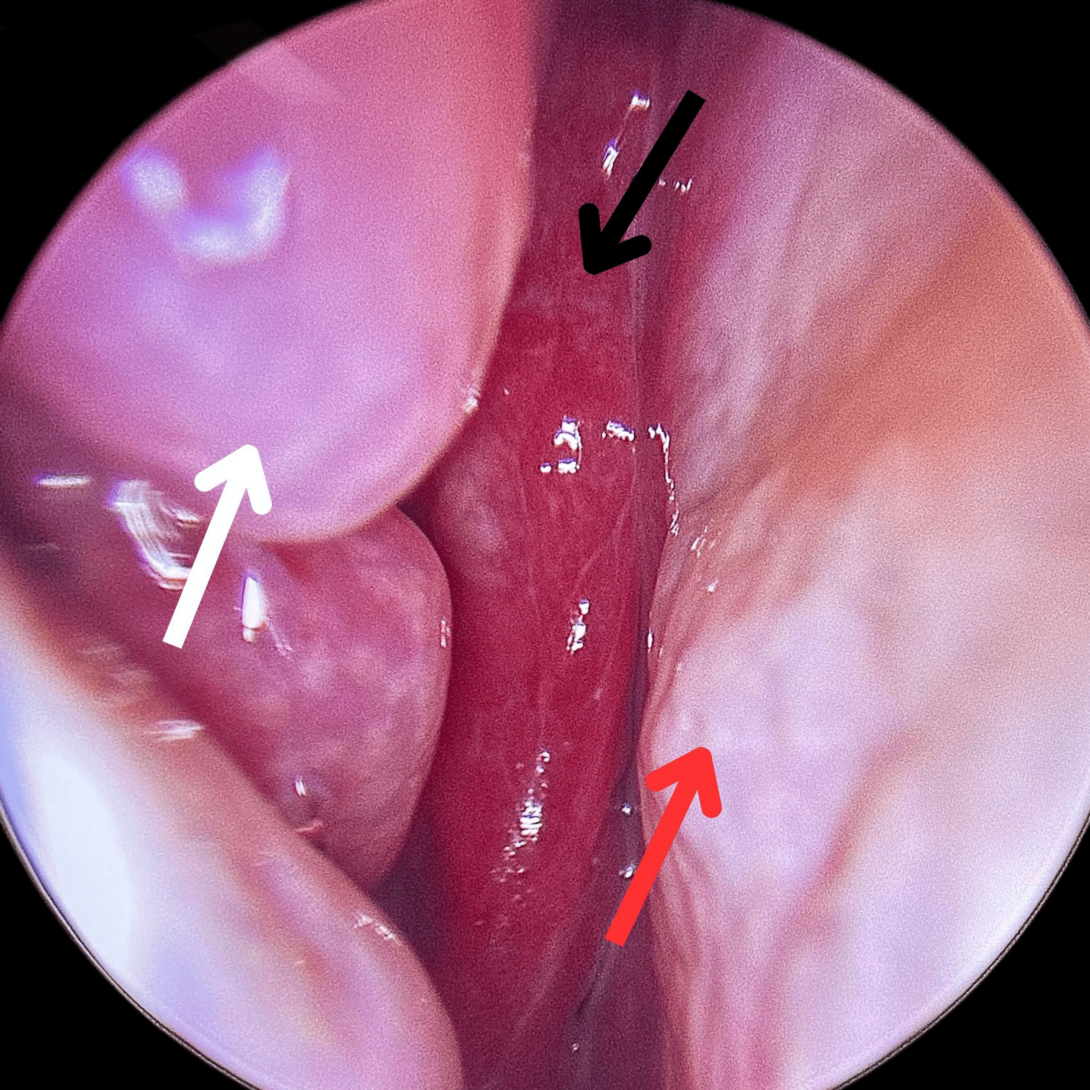
Pre-operative endoscopic image of the right nasal cavity showing the paradoxical middle turbinate (white arrow), Glomangiopericytoma (black arrow), and the nasal septum (red arrow).

Endoscopic intranasal examination revealed a right-sided, reddish, lobulated mass (Figure 1). The vascularized mass was noticed at the level of the middle turbinate, originating superiorly from the ethmosphenoidal recess and extending posteriorly into the nasopharynx (Figure 1).

**Figure 2:**
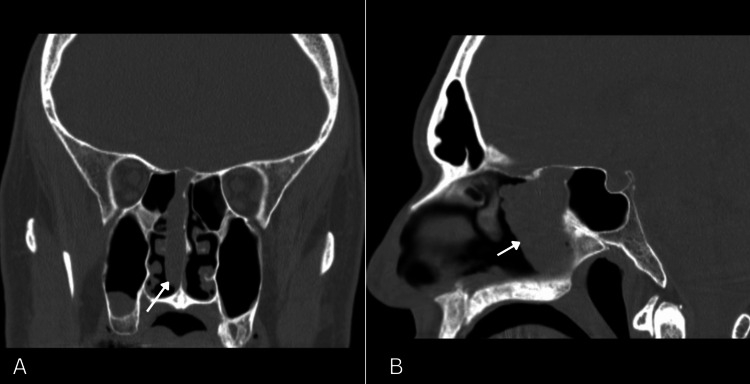
CT head imaging showing a polyp-like mass (white arrow) with indistinct separation from the middle turbinate, involving the skull base and extending inferiorly within the right nasal cavity. A. Coronal view CT head. B. Sagittal view CT head.

**Figure 3:**
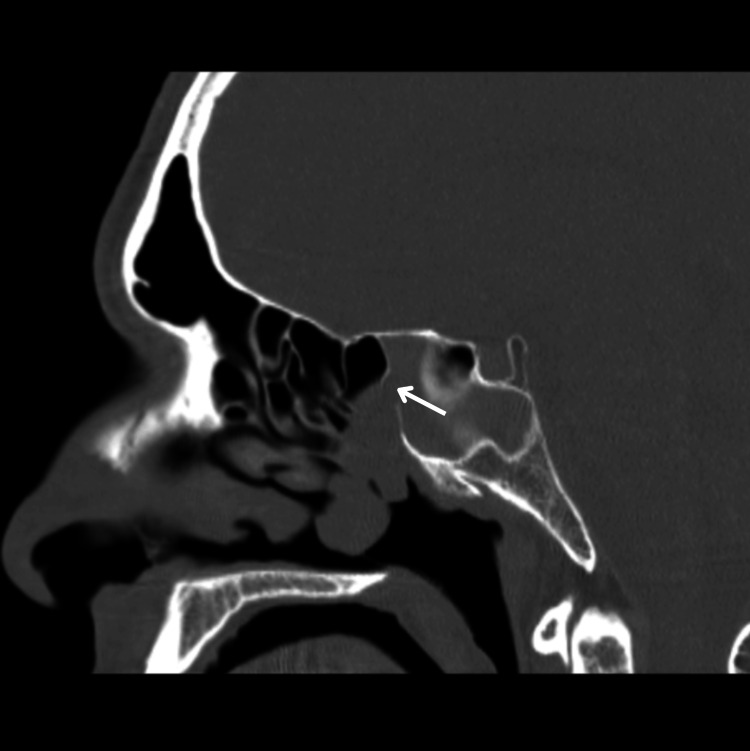
CT head (sagittal view) showing an obliterated right sphenoid sinus ostium (white arrow).

**Figure 4:**
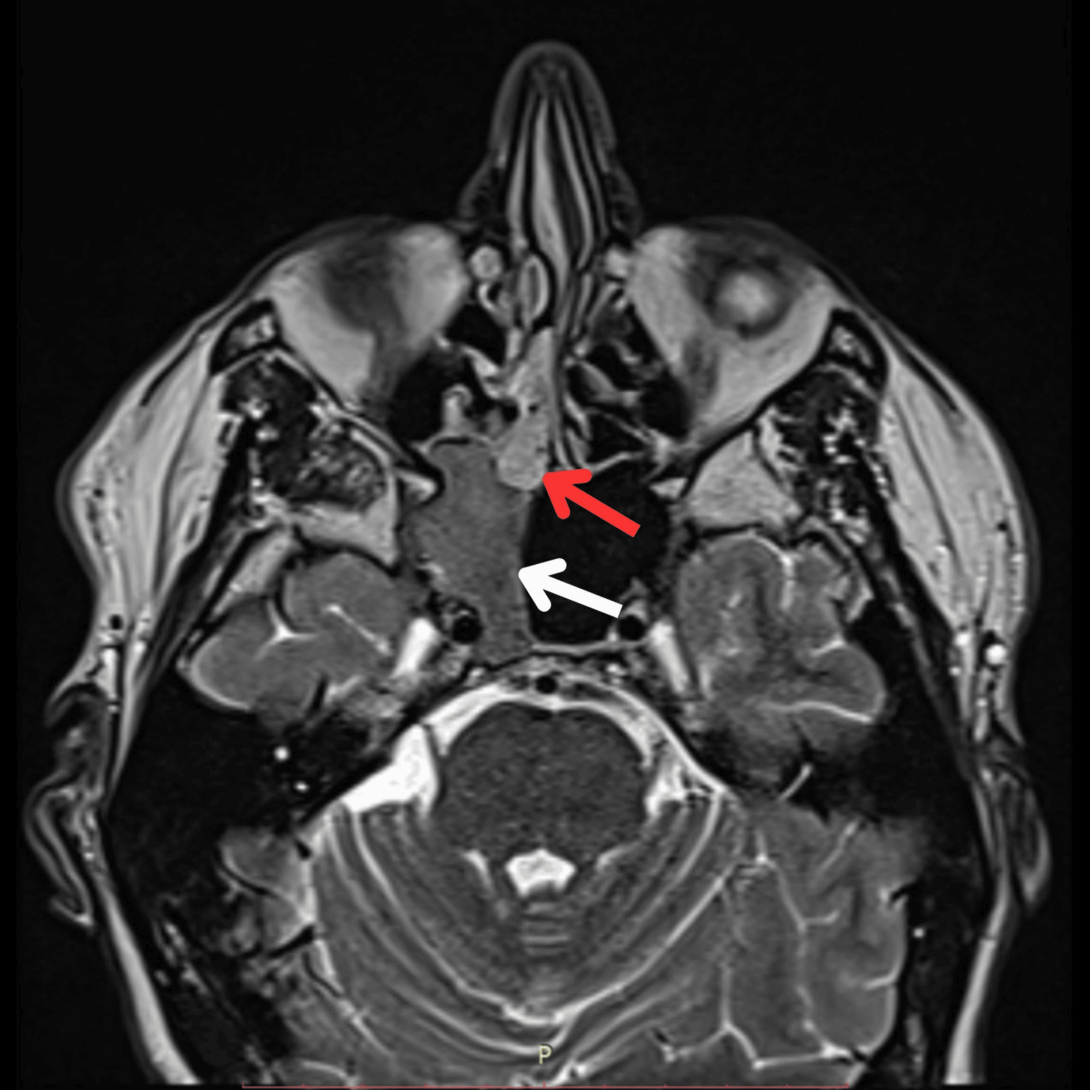
MRI head (axial view) showing the right-sided glomangiopericytoma (red arrow) and a complete opacification of right sphenoid sinus cavity with homogenous material that is isointense on T1-weighted images, illustrating a mucocele (white arrow).

Computed tomography (CT) revealed a soft tissue mass, originating from the right ethmosphenoidal recess and extending inferiorly with indistinct separation from the right middle turbinate; primarily resembling a nasal polyp (Figure 2). In addition, it showed an obliterated right sphenoid sinus ostium, consequently disrupting its drainage and aeration (Figure 3). Importantly, there was no sclerotic thickening in the sphenoid sinus walls. On the other hand, magnetic resonance imaging (MRI) revealed full opacification of the right sphenoid sinus that was isodense in T1-weighted images, illustrating a mucocele (Figure 4).

**Figure 5:**
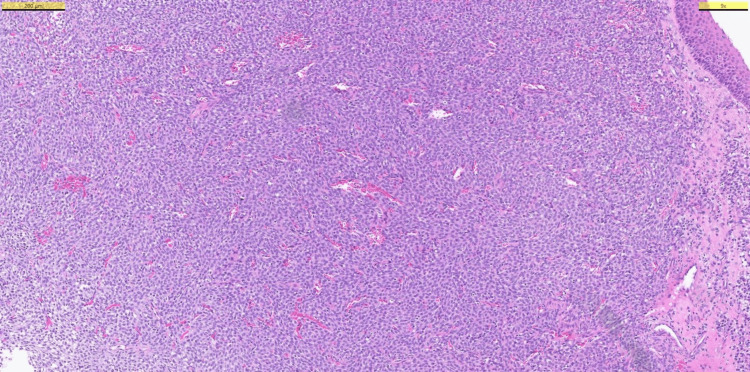
Hematoxylin and eosin (H&E) staining showing a diffuse, patternless cellular proliferation with focally fascicular and storiform patterns.

**Figure 6:**
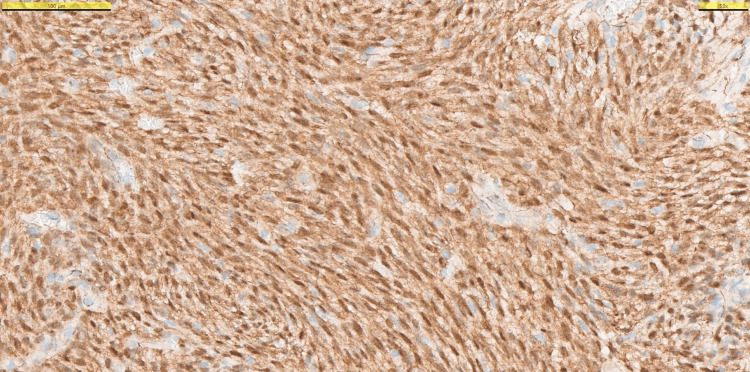
Immunohistochemistry showing a strong immunoreactivity for nuclear β-catenin.

Based on these findings, the patient was referred to a tertiary center for endonasal endoscopic resection. Endoscopic right-sided sphenoidectomy and complete resection of the mass were completed, which was sent for histopathological investigation. Immunohistochemistry revealed a patternless cellular proliferation, fascicular and storiform patterns, along with a strong expression of β-catenin (Figures 5-6). The surgical margins were clear. Ultimately, a diagnosis of skull base GLC was made. Post-operatively, the patient experienced marked improvement in olfaction and complete resolution of headaches, epistaxis, and nasal discharge at 1, 6, and 10 months of follow-up.

Our patient had a significant past medical history relevant to his condition. This includes a five-year intranasal topical corticosteroid use and right-sided balloon sinuplasty due to his chronic sinusitis. Importantly, he mentioned undergoing recurrent nasopharyngeal swabs for COVID-19 antigen testing weekly since the start of the pandemic due to continuous traveling for his occupation. He has been found to suffer from uncontrolled hypertension in the past two years.

## Discussion

Nasal cavity masses can present with non-specific, common, and mild symptoms, hiding a possible insidious cause that might need prompt surgical care. This not only prevents accurate diagnosis but also leads to a delayed or inappropriate treatment. GLC is a sinonasal tumor with a rare incidence rate, comprising only 0.5%; additionally, its slow but malignant growth causes misleading symptomatology [8]. Macroscopically, its reddish coloration and lobulated appearance could direct the diagnosis towards an inflammatory nasal polyp, and its histopathological separation from other vascularized mesenchymal tumors remains challenging [9]. The non-specific sinonasal symptoms, which mainly mimic those of a chronic sinusitis, can easily add to this diagnostic dilemma, making it necessary to gather more GLC cases in the literature for improved understanding of the condition’s clinical, anatomical, and histopathological behavior. Therefore, this report highlights how GLC can present with non-specific sinonasal symptoms and recurrent epistaxis, leading to misdiagnosis and delayed appropriate treatment.

The etiology of GLC remains poorly understood due to its infrequent occurrence. However, sinonasal trauma, pregnancy, hypertension, and corticosteroid use may be involved [2,4]. This could highlight the causes of inappropriate angiogenesis within the nasal cavity as potential risk factors. However, this still needs to be extensively studied. Our patient had long-term uncontrolled hypertension, which might have contributed to the pathophysiology. Consequently, uncontrolled hypertension coupled with the highly vascularized and friable GLC might have contributed to the later stages of recurrent epistaxis episodes, leading to interventions and a diagnosis.

Sinonasal trauma has been shown as a potential etiology [2,4]. However, there is no clear evidence of the type, intensity, and frequency of trauma. This case report points towards the long-term application of nasopharyngeal swabs as a potential contributor of intra-nasal trauma, as our patient had received these swabs for COVID-19 testing consistently multiple times a week for at least a year for occupational purposes. Another potential source of trauma was the balloon sinuplasty operation. These are unconfirmed hypotheses that necessitate further research. Furthermore, our patient was on a long-term intranasal topical corticosteroid therapy. The main risk associated with prolonged nasal corticosteroid spray use could be the local irritation and mucosal thinning with dryness, making the mucosa more prone to trauma [10]. The non-specific sinonasal symptoms caused by GLC might lead to a long-term nasal topical corticosteroid prescription, potentially creating a more favorable environment for GLC formation.

The treatment was achieved by endonasal endoscopic surgery, which aimed to remove the tumor with free margins; this was successful as reported by histopathology. However, although low-grade, sinonasal GLC is a malignant tumor, and needs close follow-up after a successful removal. While histopathological examination is crucial for an accurate diagnosis of sinonasal GLC, its resemblance to other vascularized mesenchymal tumors makes it a tricky diagnosis [9]. This is mainly due to GLC's histopathological features that are shared with solitary fibrous tumors, such as patternless cellular proliferation and the presence of staghorn-type, capillary-size capillaries [1,11]. Our patient’s histopathological assessment revealed capillary-sized vessels and a patternless cellular proliferation, but immunohistochemistry showed no reaction with STAT6, a nuclear protein that is heavily expressed in solitary fibrous tumors [9]. Post-operative immunohistochemical analysis of our patient’s resected tumor illustrated strong immunoreactivity for nuclear β-catenin (Figure 6). These, coupled with focally fascicular and storiform cellular patterns, aided us in the diagnosis of a sinonasal GLC (Figure 5) [1,2,9].

Anatomical features of sinonasal GLC usually involve the nasal cavity and rarely the skull base. In terms of the involvement of sinuses, Kono et al. showed that sphenoid sinus involvement is the least common and highlights the importance of gathering more GLC cases for better understanding the anatomical variances [12]. Our patient’s tumor arises from the ethmosphenoidal recess and the skull base, adding to its complex and unusual presentation (Figure 2). Another important finding is the mucocele within the right sphenoid sinus, which was detected by the MRI study (Figure 4). While mucoceles are mainly caused by obstruction of sinus ostia, there have not been any reports illustrating mucoceles as a potential sequelae of sinonasal GLC. Therefore, mucoceles and chronic infections of the sinuses should be further studied as a potential contributor to the development of GLC. Hence, this case potentially provides a valuable insight into better understanding the potential variances in presentation and consequences of GLC.

## Conclusions

Sinonasal GLC is an uncommon neoplasm of the head and neck region, often presenting with non-specific, chronic upper respiratory symptoms that can obscure its malignant nature and delay diagnosis, as demonstrated in our case report. The histopathological complexity of GLC was shown in our study and further complicates accurate diagnosis. Our report also highlights significant variability in clinical presentation and an uncommon skull base origin, both of which complicate diagnosis and management. Importantly, the concurrent finding of a sinus mucocele suggests a potential area for further research.

Given the malignant potential of GLC, it is crucial to enhance awareness among otolaryngologists regarding its insidious and variable progression. Despite its slow growth, timely diagnosis and endoscopic resection with clear tumor margins are vital. Clinicians should remain vigilant for non-specific, persistent symptoms such as epistaxis and anosmia, especially when accompanied by sinonasal polyp-like findings on endoscopy. A multidisciplinary approach is essential for the effective management of GLC cases. Moreover, risk factors including sinonasal trauma, hypertension, and corticosteroid use should raise clinical suspicion and warrant careful monitoring. Due to the limited literature on sinonasal GLC, the aggregation of complex cases is necessary to deepen our understanding of its clinical and anatomical progression, ultimately supporting the development of evidence-based diagnostic and management protocols.
